# Editorial: Enhanced learning and teaching via neuroscience

**DOI:** 10.3389/fpsyg.2023.1305618

**Published:** 2023-10-31

**Authors:** Lorna Uden, Gregory Siy Ching

**Affiliations:** ^1^School of Computing, Staffordshire University, Stoke-on-Trent, United Kingdom; ^2^Graduate Institute of Educational Administration and Policy, National Chengchi University, Taipei, Taiwan

**Keywords:** neuroscience, educational neuroscience, brain and learning, pedagogy, teaching and learning process

Human development and learning are fundamentally influenced by neural mechanisms, which are studied in neuroscience. The purpose of educational neuroscience is to translate research findings from neuroscience into educational practice and policy by understanding how education affects the brain. The goal of this emerging field is to link basic neuroscience, psychology, and cognitive science research with educational technology. In this Research Topic, nine articles are published. The first paper is Fang et al.. This opinion article discusses asymmetrical transfer as a reflection of functional lateralization in the left and right hemispheres of the brain. By utilizing this interlimb phenomenon, it may be possible to enhance the efficiency of time spent teaching and learning as a novel sport skill. The second paper is, Gholami et al.. This paper presents an empirical model of teachers' neuroplasticity knowledge, mindset, and epistemological beliefs. The study found that teachers with a higher score in the knowledge of neuroplasticity were more likely to have a growth mindset and a more sophisticated epistemological belief system. As a result of their knowledge of neuroplasticity, teachers' epistemological belief systems were also indirectly affected by their mindsets.

The third paper is, Meyerhofer-Parra and González-Martínez. It is a review of transmedia storytelling, which is found to promote learning engagement, but scaffolding is still needed to consolidate learning. Moreover, to guarantee a true participatory culture requires the integration of more elements that incorporate accessibility into didactic strategies, providing opportunities for learning across different styles.

The fourth paper is Uden et al.. Based on concepts from neuroscience, this paper presents an Integrated Science, Technology, Engineering, and Mathematics Projects Based Learning (STEM-PjBL) method for teaching physics to secondary students. The study found that the method facilitates the development of a commitment to physics as well as an interest in learning the subject in general. A key component of the guidelines is the idea to provide students with opportunities to practice the skills of problem-solving, critical thinking, and collaboration.

The fifth paper is Novak-Geiger. This paper is a study of neuromyths. It has been found that pedagogical psychology and neuroscience training can encourage the development of the ability of a student to recognize true and false statements in a variety of situations. By using intervention approaches that focus on a combination of activating rational thinking as well as non-prescriptive approaches, such as teacher professional development workshops and seminars focusing on the neuroscience of learning, it is possible to dispel beliefs in neuromyths and to establish evidence-based teaching practices in the classroom.

The sixth paper is Lu et al.. As the paper describes, neural markers are closely associated with attitudes toward foreign languages and the ways in which they contribute to performance in foreign languages. Based on the results of the study, it was found that subjective attitudes toward academic challenges have objective neural signatures and can have a significant effect on an individual's brain activity when in a task-like environment. The simple explanation for this is that there seems to be a domain-specific nature to the neural signatures of academic attitudes as opposed to those of general attitudes. As such, it is important to consider domain-specific factors when studying academic attitudes.

The seventh paper is Frei-Landau et al., the paper presents a project using digitally-delivered educational neuroscience in teacher education is examined, with the aim of gaining an understanding of the learning outcomes of such a platform in order to increase the quality of teacher education. As a result of employing a qualitative approach, the study identified four underlying learning outcomes: a better understanding of the brain-based mechanisms of neurodevelopmental disorders, enhanced empathy, a better understanding of the teachers' professional role, and pedagogical adaptations that were designed. It is important to note that this study provides a theoretical insight into some of the ways in which digitally delivered educational neuroscience serves as a tool for promoting inclusion in society.

The eighth and nineth papers are theoretical articles: Tokuhama-Espinosa and Borja and Tokuhama-Espinosa et al.. Using neuroconstructivism and the hierarchy of learning trajectories as a starting point, the eighth paper reexamines these theories and combines them with psychology and the way human beings interact during the teaching-learning process to suggest that radical neuroconstructivism be viewed as the framework by which teachers' professional development should take place. Using the radical neuroconstructivism framework, teachers may be able to make more visible the content knowledge that they acquire as part of their ongoing professional development program. The last paper proposes that explicit instruction of “mental frameworks” may help organize and formalize the instruction of thinking skills that underpin problem-solving—and by extension–that the more such models a person learns, the more tools they will have for future complex problem-solving.

In summary, the papers in this Research Topic highlight the emerging field of educational neuroscience for teaching and learning. These papers emphasize that using research from neuroscience, psychology, and cognitive science, can provides more effective teaching methods and techniques that can be used in a wide variety of educational settings to improve learning by students.

## Author contributions

LU: Conceptualization, Data curation, Formal analysis, Project administration, Supervision, Validation, Writing—original draft, Writing—review & editing. GC: Conceptualization, Data curation, Formal analysis, Supervision, Validation, Visualization, Writing—original draft, Writing—review & editing.

